# Leucocytes telomere length and breast cancer risk/ susceptibility: A case-control study

**DOI:** 10.1371/journal.pone.0197522

**Published:** 2018-05-21

**Authors:** Sofia Pavanello, Liliana Varesco, Viviana Gismondi, Paolo Bruzzi, Claudia Bolognesi

**Affiliations:** 1 Unit of Occupational Medicine, Department of Cardiac, Thoracic and Vascular Sciences, University of Padova, Padova, Italy; 2 Unit of Hereditary Cancer Ospedale Policlinico San Martino, Genova, Italy; 3 Unit of Clinical Epidemiology, Ospedale Policlinico San Martino, Genova, Italy; 4 Unit of Environmental Carcinogenesis Ospedale Policlinico San Martino, Genova, Italy; University of South Alabama Mitchell Cancer Institute, UNITED STATES

## Abstract

**Background:**

Telomere length in peripheral blood leukocytes (PBL-TL) was proposed as a biomarker of cancer risk. Recent scientific evidence suggested PBL-TL plays a diverse role in different cancers. Inconsistent results were obtained on PBL-TL in relation to breast cancer risk and specifically to the presence of BRCA1 and BRCA2 mutations. The aim of the present case-control study was to analyse the correlation between family history of breast cancer or presence of a BRCA mutation and PBL-TL in the hypothesis that TL is a modifier of cancer risk.

**Methods:**

PBL-TL was measured using the real-time quantitative PCR method in DNA for 142 cases and 239 controls. All the women enrolled were characterized for cancer family history. A subgroup of 48 women were classified for the presence of a BRCA mutation. PBL-TL were summarized as means and standard deviations, and compared by standard analysis of variance. A multivariable Generalised Linear Model was fitted to the data with PBL-TL as the dependent variable, case/control status and presence of a BRCA/VUS mutation as factors, and age in 4 strata as a covariate.

**Results:**

Age was significantly associated with decreasing PBL-TL in controls (p = 0.01), but not in BC cases. The telomere length is shorter in cases than in controls after adjusting for age. No effect on PBL-TL of BMI, smoke nor of the most common risk factors for breast cancer was observed. No association between PBL-TL and family history was detected both in BC cases and controls. In the multivariate model, no association was observed between BRCA mutation and decreased PBL-TL. A statistically significant interaction (p = 0.031) between case-control status and a BRCA-mutation/VUS was observed, but no effect was detected for the interaction of cancer status and BRCA or VUS.

**Conclusion:**

Our study fails to provide support to the hypothesis that PBL-TL is associated with the risk of hereditary BC, or that is a marker of inherited mutations in BRCA genes.

## Introduction

Genomic instability and in particular its most common form chromosomal instability (i.e. structural or numerical chromosome aberrations) is thought to be an early event and driving force in tumorigenesis [1,2]. Telomeres play a key role in the maintenance of chromosome integrity and stability. Telomere dysfunction has emerged as having a causative role in carcinogenesis by promoting genetic instability [3]. An essential mechanism for chromosome integrity is represented by the coverage of their end provided by telomeres. In fact, in their absence, with each cell division genetic materials would be lost [4].

Telomeres are functional complexes formed by repetitive sequences of DNA and proteins (shelterin) but their exact function and regulation have only recently begun to be understood [5–7]. Telomere length (TL) maintenance is a complex process controlled by a large number of different telomere binding proteins, some of which commonly involved in DNA repair, such as BRCA1 and BRCA2 [8–11]. TL in proliferating tissues progressively shortens with each division of somatic cells and TL in peripheral blood leucocytes (PBL) is considered a marker of biological age [12]. TL is strongly correlated across tissues [13]. Telomeres shorten at equivalent rates in somatic tissues of adults and PBL-TL is considered an useful surrogate for the other tissues.

Correlations between shortened telomeres and aging related diseases in humans such as cardiovascular diseases [14, 15], cognitive decline [16, 17], liver cirrhosis [18], premature aging syndromes [19] and also cancer have been reported.

The association of cancer risk with mean PBL-TL has been evaluated in a large number of epidemiological retrospective [20–27] and prospective [27–32] studies on different types of cancers, but the results are still inconclusive. In 2011 two meta-analyses [33, 34] provided suggestive evidence for an association between short TL and overall cancer risk. However the evidence that the odd ratios for retrospective studies were much higher than for the prospective studies suggested the presence of reverse causation bias and possible contribution of cancer therapy prior to sample collection. A recently published prospective study on almost 50,000 subjects followed for 20 years didn’t find any association between telomere length and cancer risk, while it showed a reduced survival after cancer associated with a short telomere length [35]. A recent meta-analysis pooling 62 population studies observed a non- significant association between TL shortening and overall cancer risk, and for some cancers, including gastrointestinal tumors and head and neck cancers, a significant association of short telomeres with an increased risk was observed, suggesting that telomeres may play a diverse role in different cancers [36].

A recent Mendelian randomization study, using germ line genetic variants as variables for telomere length, confirms that longer telomeres reduce risk for some non-neoplastic diseases, but increase risk for some cancers with a large variability across cancer types and tissues [37]

Breast cancer (BC) is the most common cancer and the leading cause of death from cancer in women worldwide. While most BC are sporadic in nature, approximately 5–10% are attributed to genetics, arising from autosomal dominant mutations in specific cancer genes, the strongest of which are the two breast cancer susceptibility genes BRCA1 and BRCA2 (collectively named “BRCA”). Women who carry a BRCA mutation have up to an 80% lifetime risk of developing breast cancer [38]. BRCA1 and BRCA2 proteins, which are involved in repair of DNA double strand breaks, are localized at telomeres and may regulate TL and stability [9, 10]. In addition, BRCA2 was recently described to be implicated in telomere replication [39]. Several retrospective [40–44] and prospective [27, 44–47] case-control studies examined PBL-TL in relation to breast cancer risk but only few of them[48–51] assessed this relationship in women screened for BRCA mutations. Interpretation of the findings of these studies was hampered by the inconsistency of their results. The largest of these studies (BRCA1 (n = 1,628) and BRCA2 (n = 1,506) mutation carriers) of Pooley et al. [50] found that mutation carriers (regardless of whether cancer-affected or unaffected) have longer telomeres than individuals from the same families without mutations.

Based on this background, the aims of the present study were to provide evidence on the association between family history of BC or presence of a BRCA mutation and PBL-TL in the hypothesis that telomere length is a modifier of cancer risk.

To these aims we measured PBL-TL in cases and controls subjects characterized for family history of BC and for the presence of BRCA mutations.

## Material and methods

### Study subjects

The subjects considered in this study were a subset from a larger population enrolled to evaluate the association between the presence of a pathogenic mutation in either or both the BRCA genes and increased MN frequencies [52]. The subjects were recruited in IRCCS AUO San Martino- IST- Istituto Nazionale Ricerca sul Cancro, Genova, Italy among: a) patients and their relatives referring for a genetic consultation to the Hereditary Cancer Unit; b) women attending the Radiology Unit for mammography and c) Breast cancer (BC) cases attending the Oncology Department for follow up. The entire study population includes 381 women recruited in seven years (January 2006-May 2012) with 142 cases and 239 controls. All women aged 18–80 years, with and without BC, consenting to provide a blood sample and information on their BC family history, were considered eligible for this study. Women with other cancers or with known or suspected hereditary cancer syndromes other than Hereditary Breast Ovarian Cancer were excluded. BC patients were enrolled only if they had not received chemo- or radio-therapy within 12 months before blood sampling.

The obvious bias deriving from the setting where cases and controls were selected affects the relative frequency of women with a positive family history of BC and/or a BRCA mutation, but does not affect the endpoint of our study, the PBL-TL measurement in different groups of women.

All women in the study were informed on the aims and methods of the study and gave a written consent to the use for study purposes of the clinical and family history information and to provide a blood sample which was drawn after provision of the written consent.

Detailed cancer family history information was collected by means of a personal interview with pedigree reconstruction up to three generations on both sides of the family. In all analyses, only women with BC in a 1^st^ or 2^nd^ degree relative were considered to have a positive family history.

During the study period, BRCA genetic testing was proposed to women according to protocols currently in use at the Hereditary Cancer Unit, and no test was proposed as a consequence of participation to the study. Accordingly, the results of BRCA tests were abstracted from clinical records. BRCA variant classification followed international rules [53] and variants were distinguished in three classes:—pathogenic;—uncertain significance (VUS);—not pathogenic or of no clinical significance.

### Ethics statement

All research was carried out in compliance with the Helsinki Declaration.The protocol of the study and the consent procedure were approved by the local Ethical Committee of the National Cancer Research Institute of Genoa (N. ECE08.001, 07/04/08). From all participants, informed written consent to the study was obtained and recorded.

### PBL-TL measurement by quantitative PCR

DNA was extracted from peripheral blood sample using the QIAmp DNA Mini Blood extraction Kit (Qiagen, Hiden Germany). DNA to evaluate TL was available for 142 cases and 239 controls. TL was measured in PBL DNA using the real-time quantitative PCR method developed by Cawthon [54] and described previously [55]. This method measures the relative telomere length in genomic DNA by determining the ratio of telomere repeat copy number (T) to single copy gene (S) copy number (T:S ratio) in experimental samples relative to the T/S ratio of a reference pooled sample [55]. The single-copy gene used in this study was human β-globin (*hbg*). A “seven-point” standard curve was generated from a serially diluted DNA pool (obtained from 50 DNA samples randomly selected from the case and control samples tested in the present study), ranging from 20 to 0.31 ng in each plate, so that relative quantities of T and S (in nanograms) could be determined from it. All samples and standards were run in triplicate and the average of the 3 *T/S* ratio measurements was used in the statistical analyses. The PCR runs were conducted in triplicate on a SteponePlus Real Time PCR System (Applied Biosystems). After PCR amplification, the specificity of the product was confirmed by dissociation curve analysis. To test the reproducibility of telomere length measurements, we amplified telomere (T) and *hbg* (S) in 15 samples that were replicated 3 times on each of 3 different days. The within-sample CV for the average T/S ratio over the 3 consecutive days was 8.5%, which was similar to the CV reported for the original protocol [54].

### Statistics

PBL-TL in the various subgroups were summarized as means and standard deviations, and compared by standard analysis of variance. In order to remove the confounding effect of differences in age between breast cancer cases and controls, and between subjects with and without BRCA mutations, and to assess the presence of interactions between these two variables, a multivariable Generalised Linear Model was fitted to the data with PBL-TL as the dependent variable, case/control status and presence of a BRCA/VUS mutation as factors, and age in 4 strata as a covariate. The coefficients estimated by the model should be interpreted as an adjusted difference, that is, as the weighted average difference in PBL-TL between subjects with and without each factor, estimated across strata of the other factors included in the model, with size of each stratum as weight. All p values are 2-sided. IBM SPSS, version 20 was used for all calculations.

## Results

### Characteristics of the study subjects

The details of the study population are described in Table 1.

**Table 1:** Characteristics of study subjects.

	BC Cases N (%)	Controls N (%)	Total N. (%)
Total	142 (100)	239 (100)	381 (100)
Age			
mean (range)	56 (34–79)	48 (18–72)	51 (18–79)
<40	9 (6.4)	53 (22.2)	62 (16.3)
40–55	49 (34.5)	126 (52.7)	175 (45.9)
55–65	57 (40.1)	43 (18.0)	100 (26.2)
>65	27 (19.0)	17 (7.1)	44 (11.6)
Family history			
1st degree	55 (38.8)	85 (35.6)	140 (36.7)
2nd	26 (18.3)	49 (20.5)	75 (19.7)
Neg/>2nd	61 (42.9)	105 (43.9)	166 (43.6)
BRCA classes			
Pathogenic variant BRCA1	12 (8.5)	8 (3.3)	20 (5.3)
Pathogenic variant BRCA2	5 (3.5)	3 (1.2)	8 (2.1)
VUS BRCA1	5 (3.5)	2 (0.8)	7 (1.8)
VUS BRCA2	10 (7.0)	3 (1.2)	13 (3.4)
Negative test	43 (30.3)	29 (12.1)	72 (18.9)
NA	67 (47.2)	194 (81.3)	261 (68.5)

NA not available

Cases were older than controls (mean age 56, range 34–79 and mean age 48, range 18–72 years, respectively) Due to the recruitment of a large number of subjects in the Hereditary Cancer Unit, the proportion of women with a positive 1^st^/2^nd^ degree family history of BC was high but not significantly different between BC cases and controls. A higher proportion of BC cases than controls was evaluated for the presence of BRCA mutations (75/142, 52,8% vs 45/239, 19.0%).

### PBL-TL by case-control status

PBL-TL in the total study population and in subgroups of subjects by case-control is reported in Table 2.

**Table 2:** Effect of covariates on PBL-TL by case-control status.

	BC cases		Controls	
	N (%)	Telomere lenght Mean (S.E.)	N (%)	Telomere lenght Mean (S.E.)
Comparison between cases and controls				
Crude	142	2.33 (0.06)	239	2.22 (0.05)
P value	0.189			
Adjusted for age	142	2.16 (0.057)	239	2.36 (0.069)
P value	0.025			
Age				
<40	9 (6.4)	2.14 (0.32)	53 (22.2)	2.50 (0.11)
40–55	49 (34.5)	2.42 (0.12)	126 (52.7)	2.21 (0.07)
55–65	57 (40.1)	2.32 (0.09)	43 (18.0)	2.14 (0.11)
>65	27 (19.0)	2.23 (0.13)	17 (7.1)	1.67 (0.12)
P value		0.658		0.01
BMI				
<20	13 (9.1)	2.49 (0.19)	32 (13.4)	2.32 (0.15)
20–24	58 (40.8)	2.20 (0.10)	117 (48.9)	2.17 (0.07)
25–29	25 (17.6)	2.27 (0.15)	52 (21.7)	2.15 (0.10)
30+	15 (10.5)	2.48 (0.19)	11 (4.6)	2.04 (0.18)
P value		0.462		0.673
Age at menarche				
<12	23 (16.2)	2.30 (0.18)	58 (24.3)	2.29 (0.11)
12	34 (23.9)	2.28 (0.12)	62 (25.9)	2.08 (0.07)
13+	56 (39.5)	2.31 (0.10)	94 (39.3)	2.18 (0.08)
P value		0.980		0.304
Age at 1st pregnancy				
Nulliparous	27 (19.0)	2.06 (0.15)	56 (23.4)	2.34 (0.11)
<25	32 (22.5)	2.16 (0.12)	45 (18.8)	2.09 (0.09)
25–30	47 (33.1)	2.38 (0.10)	63 (26.4)	2.19 (0.10)
>30	36 (25.4)	2.63 (0.14)	75 (31.4)	2.23 (0.09)
P value		0.017		0.439
N.of Children				
Nulliparous	27 (19.0)	2.06 (0.15)	56 (23.5)	2.34 (0.11)
1	57 (40.1)	2.48 (0.11)	77 (32.2)	2.23 (0.09)
2	45 (31.7)	2.24 (0.12)	93 (38.9)	2.19 (0.08)
3+	13 (9.2)	2.55 (0.21)	13 (5.4)	1.85 (0.17)
P value		0.078		0.215
Menopausal status				
Premenopausal	29 (20.4)	2.43 (0.17)	147 (61.5)	2.28 (0.06)
Postmenopausal	113 (79.6)	2.31 (0.07)	92 (38.5)	2.13 (0.08)
P value		0.460		0.137
Smoking Status				
Never	66 (46.5)	2.25 (0.09)	113 (47.2)	2.26 (0.07)
Current	21 (14.8)	2.51 (0.18)	53 (22.2)	2.20 (0.13)
Ex smoker	26 (18.3)	2.23 (0.15)	47 (19.7)	1.96 (0.08)
P value	29 (20.4)	0.433		0.013
Oral Contraceptive use				
Ever	61 (43.0)	2.42 (0.18)	134 (56.0)	2.23 (0.06)
Never	53 (37.3)	2.18 (0.10)	80 (33.5)	2.10 (0.09)
P value		0.111		0.223
Hormone Replacement therapy				
Ever	15 (10.6)	2.42 (0.13)	32 (13.4)	2.03 (0.12)
Never	97 (68.3)	2.28 (0.08)	180 (75.3)	2.21 (0.06)
P value		0.503		0.213
Family history				
1st degree	55 (38.8)	2.27 (0.10)	85 (35.6)	2.31 (0.09)
2nd	26 (18.3)	2.37 (0.16)	49 (20.5)	2.28 (0.10)
Neg/>2nd	61 (42.9)	2.37 (0.10)	105 (43.9)	2.13 (0.08)
P value		0.754		0.224

**Fig 1:**
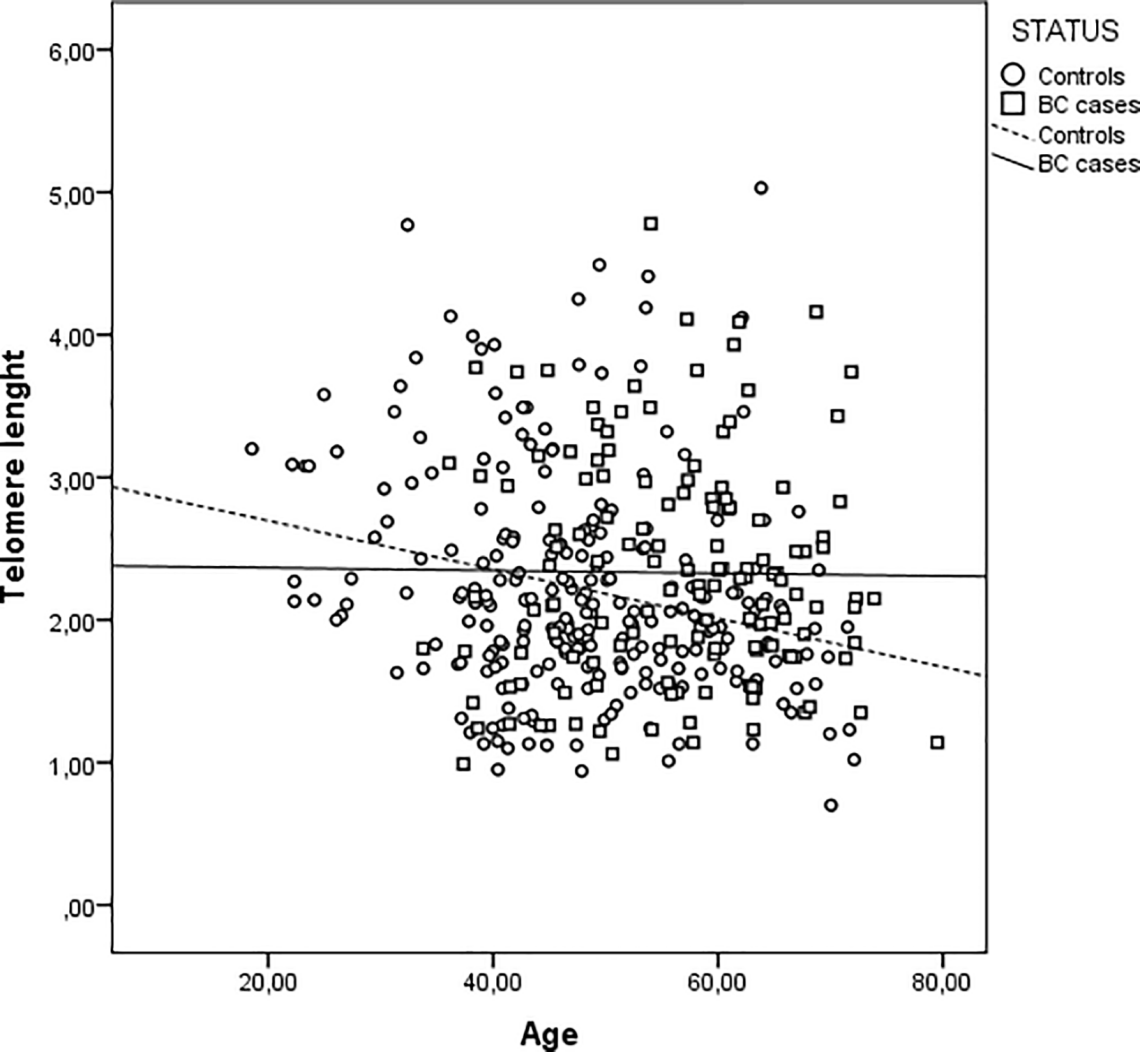
Leucocyte Telomere length (PBL-TL) as a function of age in years at blood sampling in controls and breast cancer (BC) patients. Footnote: Control: R^2^ linear = 0.059, BC cases: R^2^ linear = 1.409 E-4.

PBL-TL decreases with age in controls (p = 0.01), but not in cases (Table 2 and Fig 1).

The analysis of crude data didn’t show any difference in PBL-TL between BC cases and controls. However, mean age-adjusted PBL-TL was lower in cases compared with controls (Mean (S.E.): 2.16(0.06) vs 2.36 (0.07) p = 0.025). PBL-TL was positively associated with age of first pregnancy (p = 0.017) in BC cases. No association between PBL-TL and BMI, age at menarche, number of children, smoking habits, oral contraceptive use and hormone replacement therapy both in BC cases and in controls was detected (Table 2)

### PBL-TL by family history and BRCA status

No association between PBL-TL and family history was observed both in BC cases (p = 0.754) and controls (p = 0.224) (Table 2). In Table 3 BC cases and controls were also classified according to the presence of a pathogenic mutation or a variant of uncertain significance (VUS) in the BRCA1 or BRCA2 gene. Among subjects tested negative for a mutation in either gene, mean PBL-TL was similar in cases and controls (mean (SE) = 2.58 (0.12) and 2.42 (0.15)), respectively. Among controls, no significant variation in mean PBL-TL with the BRCA mutation status was observed. The lowest mean PBL-TL value was detected in VUS BRCA2 variants (3subjects) ((Mean (S.E.): 2.19(0.19)) and the highest mean TL value was observed in VUS BRCA1 variants (2 subjects) ((Mean (S.E.): 3.36 (0.23). Among cases, significant variation in mean PBL-TL with the BRCA mutation status was observed (p = 0.006). Among BC cases the lowest mean PBL-TL value was detected in VUS BRCA2 variants (10 subjects) (Mean (S.E.): 1.78 (0.19)) no significant variation in mean PBL-TL was seen in patients tested positive for the presence of BRCA pathogenic mutations (mean (SE) = 1.94 (0.17), p = 0.397) (Table 3)

**Table 3:** Effect of BRCA status/classes on PBL-TL in cases and controls.

	PBL-LTL (T/S)					
BRCA STATUS	Cases			Controls		
	N	Mean	SE*	N	Mean	SE
Total	75	2.27	.096	45	2.44	.11
negative test	43	2.58	.13	29	2.42	.16
BRCA1	12	1.95	.23	8	2.23	.21
BRCA2	5	1.92	.27	3	2.89	.30
VUS BRCA1	5	1.79	.30	2	3.36	.23
VUS BRCA2	10	1.78	.20	3	2.19	.19
P		0.006			0.284	
BRCA	17	1.94	.17	11	2,40	0.16
P		0.			0.352	

*Standard error

Finally, BRCA/VUS mutation and cancer/control status were included in a Multivariable Generalized Linear Model as Factors, with age in 4 strata as a covariate (Table 4). The presence of an interaction (synergism) between each pair of them on PBL-TL was evaluated. Case-control status and age were not significantly associated with PBL-TL (p = 0.113 and p = 0.356, respectively). In the multivariate model, the presence of a BRCA mutation was not associated with a decreased PBL-TL (adjusted difference = -0.339, p = 0.112). A statistically significant interaction between case-control status and a BRCA-mutation/VUS was observed (p = 0.031), but no reduction of PBL-TL was observed when the interaction between cancer status was evaluated for BRCA and VUS mutations. No interaction was seen between age and case-control status or BRCA mutation status.

**Table 4:** Association between presence of a pathogenic mutation or variant of unknown significance (VUS) in BRCA1-2 genes and PBL-TL: Multivariate linear regression analysis.

Parameter	Adjusted Difference	S.E.	95% C.L.	F (df)	P
Case/Control Status					
Breast Cancer vs Controls	0.313	0.196	-0.075–0.702	2.549 (1)	0,113
BRCA Mutation Status					
Mutated vs negative	-0.339	0.175	-0.685–0.008	2.235 (2)	0.112
VUS vs negative	-0.273	0.219	-0.706–0.160		
AGE					
<40 vs 40–55 vs 55–65 vs >65	-0.078	0.084	-0.245–0.089	0.860 (1)	0.356
Interaction between CC-status -BRCA^a^ CC- mutational status CC-VUS	0.586 1.048	0.351 0.437	-0.109–1,280 0.182–1.914	3.568(2)	0.031
Interaction AGE-BRCA^b^	-	-	-	1.372 (2)	0.258
Interaction CC-Status Age^b^	-	-	-	2.083 (1)	0.152

^a^ See Methods and Results for the explanation of the meaning and for the interpretation of the interaction coefficient

^b^ Excluded from the final model

## Discussion

The aim of our study was to evaluate the possible association between PBL-TL and the risk factors for breast cancer, mainly focusing on familiar and genetic factors (i.e. BRCA mutations). PBL-TL was measured in a group of healthy women and breast cancer patients with and without family history and in a subgroup of women evaluated for the presence of BRCA mutations.

Shorter PBL-TL was observed in our study in BC cases with respect to controls after adjusting for age. Several retrospective [40–44] and prospective [27; 44–47] case-control studies have examined PBL-TL in relation to breast cancer risk. The substantial inconsistency among the results of these studies can be accounted by a number of factors including the study design (prospective or retrospective), the use of different methods to measure TL and the potential exposure to genotoxic agents for diagnostic or therapeutic purposes. However, a strong association between shorter PBL-TL and BC risk was recently observed in a large retrospective study (2243 cases and 2181 controls) [44]. This suggests that telomere shortening may occur after diagnosis, possibly as a consequence of diagnostic and therapeutic procedures, and therefore, may not be of value in cancer prediction.

The main covariates that were found in previous studies to be associated with PBL-TL were analyzed in the present study. In agreement with what previously found [12] also in our study increasing age was significantly associated with decreasing PBL-TL of controls, while no effect of age on PBL-TL was detected in BC cases. This result is in agreement with the data reported in studies on cancer patients where the effect of age on TL is undetectable or negligible, due to the complex alterations in telomere maintenance mechanisms associated with the carcinogenic process, diagnostic and therapeutic procedures. No effect of BMI on PBL-TL was observed both in controls and in BC cases tested in our study. Contrasting results were reported on the role of BMI on PBL-TL, although TL shortening associated with increasing BMI was described in large size studies and in a recent meta-analysis [56].

The influence of cigarette smoking was not detected in our study in contrast with some but not all previous studies [57, 58].

No association was found between PBL-TL and the most common risk factors for breast cancer considered in our study, such as age at menarche, age of first pregnancy, number of children, use of oral contraceptive, hormone replacement therapy.

The main objective of our study was to investigate the potential association between PBL-TL and familiar factors. All the women enrolled were characterized for cancer family history, considering women with BC in a 1st or 2nd degree relative to have a positive family history. No difference in PBL-TL was observed with respect to family history both in BC cases and in controls. The large majority of studies on PBL-TL in BC didn’t characterize the patients for family history. Our results are at variance with those of a study focused on hereditary BC that reported a significantly shorter PBL-TL in familial BC patients than in the control population and shorter PBL-TL associated with earlier onset of BC in successive generations of affected families [48].

The association between BRCA mutation status and PBL-TL was also investigated in our study in a subgroup of subjects for which data on genetic status were available. A statistically significant association between presence of a pathogenic mutation in the BRCA genes and shorter PBL-TL was observed in BC patients but not in controls. The results are difficult to interpret, because the evidence from the literature on the PBL-TL in BRCA mutation carriers is again conflicting. A number of studies reported shortened telomeres in leucocytes [48] or didn’t find difference [49] in TL in BRCA mutation carriers with respect to normal populations. Recently a large study showed that BRCA mutation carriers have longer telomeres than non-mutation carriers regardless of cancer status [50]. Our results suggest that the difference may be due to diagnostic or therapeutic procedures in hereditary BC cases rather than to the genetic alterations.

A common bias of the studies on cancer patients, including ours, is that the enrollment is usually carried out after the diagnosis and the measurement of the PBL-TL could be affected by the diagnosis or treatment. It was been shown that chemotherapy exerts a transient telomere shortening effect [59] and telomere loss have been reported in BC patients following radiotherapy [60]. Despite our efforts to exclude patients recently treated with antiblastic drugs or radiations we can’t rule out the effects of exposures associated with chromosome instability and telomere loss in cancer patients recruited in our study.

In conclusion, our study fails to provide support to the hypothesis that PBL-TL is associated with the risk of hereditary BC, or that is a marker of inherited mutations in BRCA genes, suggesting instead that some of the correlations reported in the past may be due to the confounding effect of the procedures carried out in BC patients after diagnosis.

## References

[1] Michor F. Chromosomal instability and human cancer. Philos Trans R Soc Lond B Biol Sci. 2005;360(1455):631–5. doi: 10.1098/rstb.2004.1617 15897185 10.1098/rstb.2004.1617 PMC1569472

[2] Negrini S, Gorgoulis VG, Halazonetis TD.Genomic instability—an evolving hallmark of cancer. Nat Rev Mol Cell Biol. 2010;11(3):220–8. doi: 10.1038/nrm2858 20177397 10.1038/nrm2858

[3] Feldser DM, Hackett JA, Greider CW. Telomere dysfunction and the initiation of genome instability. Nat Rev Cancer. 2003;3(8):623–7. doi: 10.1038/nrc1142 12894250 10.1038/nrc1142

[4] Moyzis RK, Buckingham JM, Cram LS, Dani M, Deaven LL, Jones MD et al A highly conserved repetitive DNA sequence, (TTAGGG)n, present at the telomeres of human chromosomes. Proc Natl Acad Sci U S A. 1988;85(18):6622–6. 3413114 10.1073/pnas.85.18.6622 PMC282029

[5] Armanios M, Blackburn EH. The telomere syndromes. Nat Rev Genet. 2012;13(10):693–704. doi: 10.1038/nrg3246 22965356 10.1038/nrg3246 PMC3548426

[6] Lin J, Kaur P, Countryman P, Opresko PL, Wang H. Unraveling secrets of telomeres: One molecule at a time. DNA Repair (Amst). 2014;20:142–53. 24569170 10.1016/j.dnarep.2014.01.012 PMC4833095

[7] Boccardi V, Poalisso G. Telomerase activation: a potential key modulator for human healthspan and longevity. Ageing Res Rev. 2014;15:1–5. doi: 10.1016/j.arr.2013.12.006 24561251 10.1016/j.arr.2013.12.006

[8] Webb CJ, Wu Y, Zakian VA. DNA repair at telomeres: keeping the ends intact. Cold Spring Harb Perspect Biol. 2013;5(6): doi: 10.1101/cshperspect.a012666.2013;5(6) 10.1101/cshperspect.a012666 PMC3660827 23732473

[9] Min J, Choi ES, Hwang K, Kim J, Sampath S, Venkitaraman AR et al The breast cancer susceptibility gene BRCA2 is required for the maintenance of telomere homeostasis. J Biol Chem. 2012;287(7):5091–101. doi: 10.1074/jbc.M111.278994 22187435 10.1074/jbc.M111.278994 PMC3281639

[10] Ballal RD, Saha T, Fan S, Haddad BR, Rosen EM. BRCA1 localization to the telomere and its loss from the telomere in response to DNA damage. J Biol Chem. 2009;284:36083–98. doi: 10.1074/jbc.M109.025825 19797051 10.1074/jbc.M109.025825 PMC2794724

[11] Takubo K, Izumiyama-Shimomura N, Honma N, Sawabe M, Arai T, Kato M, et al Telomere lengths are characteristic in each human individual. Exp Gerontol. 2002;37(4):523–31. 11830355 10.1016/s0531-5565(01)00218-2

[12] Frenck RW Jr, Blackburn EH, Shannon KM. The rate of telomere sequence loss in human leukocytes varies with age. Proc Natl Acad Sci U S A. 1998;95:5607–10. 9576930 10.1073/pnas.95.10.5607 PMC20425

[13] Daniali L, Benetos A, Susser E, Kark JD, Labat C, Kimura M, et al Telomeres shorten at equivalent rates in somatic tissues of adults. Nat Commun. 2013;4:1597 doi: 10.1038/ncomms2602 23511462 10.1038/ncomms2602 PMC3615479

[14] Brouilette S, Singh RK, Thompson JR, Goodall AH, Samani NJ. White cell telomere length and risk of premature myocardial infarction. Arterioscler Thromb Vasc Biol. 2003;23:842–6. doi: 10.1161/01.ATV.0000067426.96344.32 12649083 10.1161/01.ATV.0000067426.96344.32

[15] Benetos A, Gardner JP, Zureik M, Labat C, Xiaobin L, Adamopoulos C, et al A.Short telomeres are associated with increased carotid atherosclerosis in hypertensive subjects. Hypertension. 2004;43(2):182–5. doi: 10.1161/01.HYP.0000113081.42868.f4 14732735 10.1161/01.HYP.0000113081.42868.f4

[16] Panossian LA, Porter VR, Valenzuela HF, Zhu X, Reback E, Masterman D, et al Telomere shortening in T cells correlates with Alzheimer’s disease status. Neurobiol Aging. 2003;24:77–84. 12493553 10.1016/s0197-4580(02)00043-x

[17] von Zglinicki T, Serra V, Lorenz M, Saretzki G, Lenzen-Grossimlighaus R, Gessner R, et al Short telomeres in patients with vascular dementia: an indicator of low antioxidative capacity and a possible risk factor? Lab Invest. 2000;80:1739–47. 11092534 10.1038/labinvest.3780184

[18] Wiemann SU, Satyanarayana A, Tsahuridu M, Tillmann HL, Zender L, Klempnauer J, et al Hepatocyte telomere shortening and senescence are general markers of human liver cirrhosis. FASEB J. 2002;16:935–42. doi: 10.1096/fj.01-0977com 12087054 10.1096/fj.01-0977com

[19] Blasco MA. Telomeres and human disease: ageing, cancer and beyond. Nat Rev Genet 2005;6:611–22. doi: 10.1038/nrg1656 16136653 10.1038/nrg1656

[20] Wu X, Amos CI, Zhu Y, Zhao H, Grossman BH, Shay JW, et al Telomere dysfunction: a potential cancer predisposition factor. J Natl Cancer Inst. 2003;95:1211–18. 12928346 10.1093/jnci/djg011

[21] Broberg K, Bjork J, Paulsson K, Hoglund M, Albin M. Constitutional short telomeres are strong genetic susceptibility markers for bladder cancer. Carcinogenesis. 2005;26:1263–71. doi: 10.1093/carcin/bgi063 15746160 10.1093/carcin/bgi063

[22] Shao L, Wood CG, Zhang D, Tannir NM, Matin S, Dinney CP, et al Telomere dysfunction in peripheral lymphocytes as a potential predisposition factor for renal cancer. J Urol. 2007; 178:1492–6. doi: 10.1016/j.juro.2007.05.112 17707063 10.1016/j.juro.2007.05.112

[23] Jang JS, Choi YY, Lee WK, Choi JE, Cha SI, Kim YJ, et al Telomere length and the risk of lung cancer. Cancer Sci. 2008;99:1385–9. doi: 10.1111/j.1349-7006.2008.00831.x 18452563 10.1111/j.1349-7006.2008.00831.x PMC11158548

[24] Hou L, Savage SA, Blaser MJ, Perez-Perez G, Hoxha M, Dioni L, et al Telomere length in peripheral leukocyte DNA and gastric cancer risk. Cancer Epidemiol Biomarkers Prev. 2009;18:3103–9. doi: 10.1158/1055-9965.EPI-09-0347 19861514 10.1158/1055-9965.EPI-09-0347 PMC2938741

[25] Zheng YL, Zhou X, Loffredo CA, Shields PG, Sun B. Telomere deficiencies on chromosomes 9p, 15p, 15q and Xp: potential biomarkers for breast cancer risk. Hum Mol Genet. 2011;20:378–86. doi: 10.1093/hmg/ddq461 20956286 10.1093/hmg/ddq461 PMC3005901

[26] McGrath M, Wong JY, Michaud D, Hunter DJ, De Vivo I. Telomere length, cigarette smoking, and bladder cancer risk in men and women. Cancer Epidemiol Biomarkers Prev. 2007;16:815–9. doi: 10.1158/1055-9965.EPI-06-0961 17416776 10.1158/1055-9965.EPI-06-0961

[27] Willeit P, Willeit J, Mayr A, Weger S, Oberhollenzer F, Brandstätter A, et al Telomere length and risk of incident cancer and cancer mortality. JAMA. 2010;304:69–75. doi: 10.1001/jama.2010.897 20606151 10.1001/jama.2010.897

[28] De Vivo I, Prescott J, Wong JY, Kraft P, Hankinson SE, Hunter DJ. A prospective study of relative telomere length and postmenopausal breast cancer risk. Cancer Epidemiol Biomarkers Prev. 2009;18(4):1152–6. doi: 10.1158/1055-9965.EPI-08-0998 19293310 10.1158/1055-9965.EPI-08-0998 PMC2732000

[29] Han J, Qureshi AA, Prescott J, Guo Q, Ye L, Hunter DJ, et al A prospective study of telomere length and the risk of skin cancer. J Invest Dermatol. 2009;129(2):415–21. doi: 10.1038/jid.2008.238 18668136 10.1038/jid.2008.238 PMC2632304

[30] Lee IM, Lin J, Castonguay AJ, Barton NS, Buring JE, Zee RY. Mean leukocyte telomere length and risk of incident colorectal carcinoma in women: a prospective, nested case-control study. Clin Chem Lab Med. 2010;48(2):259–62. doi: 10.1515/CCLM.2010.049 19961392 10.1515/CCLM.2010.049 PMC2818287

[31] Mirabello L, Huang WY, Wong JY, Chatterjee N, Reding D, Crawford ED, et al The association between leukocyte telomere length and cigarette smoking, dietary and physical variables, and risk of prostate cancer. Aging Cell. 2009;8(4):405–13. doi: 10.1111/j.1474-9726.2009.00485.x 19493248 10.1111/j.1474-9726.2009.00485.x PMC2742954

[32] Zee RY, Castonguay AJ, Barton NS, Buring JE. Mean telomere length and risk of incident colorectal carcinoma: a prospective, nested case control approach. Cancer Epidemiol Biomarkers Prev. 2009;18(8):2280–2. doi: 10.1158/1055-9965.EPI-09-0360 19661087 10.1158/1055-9965.EPI-09-0360 PMC2774215

[33] Ma H, Zhou Z, Wei S, Liu Z, Pooley KA, Dunning AM, et al Shortened telomere length is associated with increased risk of cancer: a meta-analysis. PLoS One. 2011;6(6):e20466 doi: 10.1371/journal.pone.0020466 21695195 10.1371/journal.pone.0020466 PMC3112149

[34] Wentzensen IM, Mirabello L, Pfeiffer RM, Savage SA. The association of telomere length and cancer: a meta-analysis. Cancer Epidemiol Biomarkers Prev. 2011;20(6):1238–50. doi: 10.1158/1055-9965.EPI-11-0005 21467229 10.1158/1055-9965.EPI-11-0005 PMC3111877

[35] Weischer M, Nordestgaard BG, Cawthon RM, Freiberg JJ, Tybjærg-Hansen A, Bojesen SE. Short telomere length, cancer survival, and cancer risk in 47102 individuals. J Natl Cancer Inst. 2013;105(7):459–68. doi: 10.1093/jnci/djt016 23468462 10.1093/jnci/djt016

[36] Zhu X, Han W, Xue W, Zou Y, Xie C, Du J, et al The association between telomere length and cancer risk in population studies. Sci Rep. 2016;6:22243 doi: 10.1038/srep22243 26915412 10.1038/srep22243 PMC4768100

[37] Telomeres Mendelian Randomization Collaboration, Haycock PC, Burgess S, Nounu A, Zheng J, Okoli GN, Bowden J, et al Association Between Telomere Length and Risk of Cancer and Non-Neoplastic Diseases: A Mendelian Randomization Study. JAMA Oncol. 2017;3(5):636–51. doi: 10.1001/jamaoncol.2016.5945 28241208 10.1001/jamaoncol.2016.5945 PMC5638008

[38] Milne RL, Antoniou AC. Modifiers of breast and ovarian cancer risks for BRCA1 and BRCA2 mutation carriers. Endocr Relat Cancer. 2016;23(10):T69–84. doi: 10.1530/ERC-16-0277 27528622 10.1530/ERC-16-0277

[39] Badie S, Escandell JM, Bouwman P, Carlos AR, Thanasoula M, Gallardo MM, et al BRCA2 acts as a RAD51 loader to facilitate telomere replication and capping. Nat Struct Mol Biol. 2010;17:1461–69. doi: 10.1038/nsmb.1943 21076401 10.1038/nsmb.1943 PMC2998174

[40] Shen J, Terry MB, Gurvich I, Liao Y, Senie RT, Santella RM. Short telomere length and breast cancer risk: a study in sister sets. Cancer Res. 2007;67(11):5538–44. doi: 10.1158/0008-5472.CAN-06-3490 17545637 10.1158/0008-5472.CAN-06-3490

[41] Svenson U, Nordfjäll K, Stegmayr B, Manjer J, Nilsson P, Tavelin B, et al Breast cancer survival is associated with telomere length in peripheral blood cells. Cancer Res. 2008;68(10):3618–23. doi: 10.1158/0008-5472.CAN-07-6497 18483243 10.1158/0008-5472.CAN-07-6497

[42] Shen J, Gammon MD, Terry MB, Wang Q, Bradshaw P, Teitelbaum SL, et al Telomere length, oxidative damage, antioxidants and breast cancer risk. Int J Cancer. 2009;124(7):1637–43. doi: 10.1002/ijc.24105 19089916 10.1002/ijc.24105 PMC2727686

[43] Gramatges MM, Telli ML, Balise R, Ford JM. Longer relative telomere length in blood from women with sporadic and familial breast cancer compared with healthy controls. Cancer Epidemiol Biomarkers Prev. 2010;(2):605–13. doi: 10.1158/1055-9965.EPI-09-0896 20142254 10.1158/1055-9965.EPI-09-0896

[44] Pooley KA, Sandhu MS, Tyrer J, Shah M, Driver KE, Luben RN, et al Telomere length in prospective and retrospective cancer case-control studies. Cancer Res. 2010;70(8):3170–6. doi: 10.1158/0008-5472.CAN-09-4595 20395204 10.1158/0008-5472.CAN-09-4595 PMC2855947

[45] De Vivo I, Prescott J, Wong JY, Kraft P, Hankinson SE, Hunter DJ. A prospective study of relative telomere length and postmenopausal breast cancer risk. Cancer Epidemiol Biomarkers Prev. 2009;18(4):1152–6. doi: 10.1158/1055-9965.EPI-08-0998 19293310 10.1158/1055-9965.EPI-08-0998 PMC2732000

[46] Kim S, Sandler DP, Carswell G, De Roo LA, Parks CG, Cawthon R, et al Telomere length in peripheral blood and breast cancer risk in a prospective case-cohort analysis: results from the Sister Study. Cancer Causes Control. 2011;22(7):1061–6. doi: 10.1007/s10552-011-9778-8 21643930 10.1007/s10552-011-9778-8 PMC3445257

[47] Qu S, Wen W, Shu XO, Chow WH, Xiang YB, Wu J, et al Association of leukocyte telomere length with breast cancer risk: nested case-control findings from the Shanghai Women's Health Study. Am J Epidemiol. 2013;177(7):617–24. doi: 10.1093/aje/kws291 23444102 10.1093/aje/kws291 PMC3657533

[48] Martinez-Delgado B, Gallardo M, Tanic M, Yanowsky K, Inglada-Perez L, Barroso A, et al Short telomeres are frequent in hereditary breast tumors and are associated with high tumor grade. Breast Cancer Res Treat. 2013;141(2):231–42. doi: 10.1007/s10549-013-2696-6 24036693 10.1007/s10549-013-2696-6

[49] Killick E, Tymrakiewicz M, Cieza-Borrella C, Smith P, Thompson DJ, Pooley KA, et al Telomere Length Shows No Association with BRCA1 and BRCA2 Mutation Status. PLoS One. 2014;9(1):e86659 doi: 10.1371/journal.pone.0086659 24489760 10.1371/journal.pone.0086659 PMC3906069

[50] Pooley KA, McGuffog L, Barrowdale D, Frost D, Ellis SD, Fineberg E, et al Lymphocyte telomere length is long in BRCA1 and BRCA2 mutation carriers regardless of cancer-affected status. Cancer Epidemiol Biomarkers Prev. 2014;23(6):1018–24. doi: 10.1158/1055-9965.EPI-13-0635-T 24642354 10.1158/1055-9965.EPI-13-0635-T PMC4266102

[51] Thorvaldsdottir B, Aradottir M, Stefansson OA, Bodvarsdottir SK, Eyfjörd JE. Telomere Length Is Predictive of Breast Cancer Risk in BRCA2 Mutation Carriers. Cancer Epidemiol Biomarkers Prev. 2017;26(8):1248–54. doi: 10.1158/1055-9965.EPI-16-0946 28235830 10.1158/1055-9965.EPI-16-0946

[52] Bolognesi C, Bruzzi P, Gismondi V, Volpi S, Viassolo V, Pedemonte S, et al Clinical application of micronucleus test: a case-control study on the prediction of breast cancer risk/susceptibility. PLoS One. 2014;9(11):e112354 doi: 10.1371/journal.pone.0112354 25415331 10.1371/journal.pone.0112354 PMC4240584

[53] Plon SE, Eccles DM, Easton D, Foulkes WD, Genuardi M, Greenblatt MS, et al IARC Unclassified Genetic Variants Working Group. Sequence variant classification and reporting: recommendation for improving the interpretation of cancer susceptibility genetic test results. Hum Mutat. 2008;29(11):1282–91. doi: 10.1002/humu.20880 18951446 10.1002/humu.20880 PMC3075918

[54] Cawthon RM. Telomere measurement by quantitative PCR. Nucleic Acids Res. 2002;30(10):e47 12000852 10.1093/nar/30.10.e47 PMC115301

[55] Pavanello S, Pesatori AC, Dioni L, Hoxha M, Bollati V, Siwinska E, et al Shorter telomere length in peripheral blood lymphocytes of workers exposed to polycyclic aromatic hydrocarbons. Carcinogenesis 2010;31(2):216–21. doi: 10.1093/carcin/bgp278 19892797 10.1093/carcin/bgp278 PMC3491668

[56] Müezzinler A, Zaineddin AK, Brenner H. Body mass index and leukocyte telomere length in adults: a systematic review and meta-analysis. Obes Rev. 2014;15(3):192–201. doi: 10.1111/obr.12126 24165286 10.1111/obr.12126

[57] Latifovic L, Peacock SD, Massey TE, King WD. The Influence of Alcohol Consumption, Cigarette Smoking, and Physical Activity on Leukocyte Telomere Length. Cancer Epidemiol Biomarkers Prev. 2016;25(2):374–80. doi: 10.1158/1055-9965.EPI-14-1364 26656293 10.1158/1055-9965.EPI-14-1364

[58] Astuti Y, Wardhana A, Watkins J, Wulaningsih W, PILAR Research Network. Cigarette smoking and telomere length: A systematic review of 84 studies and meta-analysis. Environ Res. 2017;158:480–9. doi: 10.1016/j.envres.2017.06.038 28704792 10.1016/j.envres.2017.06.038 PMC5562268

[59] Benitez-Buelga C, Sanchez-Barroso L, Gallardo M, Apellániz-Ruiz M, Inglada-Pérez L, Yanowski K, et al Impact of chemotherapy on telomere length in sporadic and familial breast cancer patients. Breast Cancer Res Treat. 2015;149(2):385–94. doi: 10.1007/s10549-014-3246-6 25528024 10.1007/s10549-014-3246-6 PMC4824277

[60] Banerjee B, Sharma S, Hegde S, Hande MP. Analysis of telomere damage by fluorescence in situ hybridisation on micronuclei in lymphocytes of breast carcinoma patients after radiotherapy. Breast Cancer Res Treat. 2008;107:25–31. doi: 10.1007/s10549-007-9530-y 17333339 10.1007/s10549-007-9530-y

